# Recent advances of nanomaterial-based anti-angiogenic therapy in tumor vascular normalization and immunotherapy

**DOI:** 10.3389/fonc.2022.1039378

**Published:** 2022-11-29

**Authors:** Mingshu Xiao, Yueli Shi, Sujing Jiang, Mengqing Cao, Weiyu Chen, Yun Xu, Zhiyong Xu, Kai Wang

**Affiliations:** Department of Respiratory and Critical Care Medicine, The Fourth Affiliated Hospital of Zhejiang University School of Medicine, Yiwu, Zhejiang, China

**Keywords:** cancer, anti-angiogenesis therapy, immunotherapy, tumor immune microenvironment (TIM), nanomedicine

## Abstract

Anti-angiogenesis therapy and immunotherapy are the first-line therapeutic strategies for various tumor treatments in the clinic, bringing significant advantages for tumor patients. Recent studies have shown that anti-angiogenic therapy can potentiate immunotherapy, with many clinical trials conducted based on the combination of anti-angiogenic agents and immune checkpoint inhibitors (ICIs). However, currently available clinical dosing strategies and tools are limited, emphasizing the need for more improvements. Although significant progress has been achieved, several big questions remained, such as how to achieve cell-specific targeting in the tumor microenvironment? How to improve drug delivery efficiency in tumors? Can nanotechnology be used to potentiate existing clinical drugs and achieve synergistic sensitization effects? Over the recent few years, nanomedicines have shown unique advantages in antitumor research, including cell-specific targeting, improved delivery potentiation, and photothermal effects. Given that the applications of nanomaterials in tumor immunotherapy have been widely reported, this review provides a comprehensive overview of research advances on nanomaterials in anti-angiogenesis therapy, mainly focusing on the immunosuppressive effects of abnormal tumor vessels in the tumor immune microenvironment, the targets and strategies of anti-angiogenesis nanomedicines, and the potential synergistic effects and molecular mechanisms of anti-angiogenic nanomedicines in combination with immunotherapy, ultimately providing new perspectives on the nanomedicine-based synergy between anti-angiogenic and immunotherapy.

## Introduction

Angiogenesis is the process of neovascularization from existing peripheral blood vessels, providing essential nutrients for solid tumor growth and a pathway for malignant metastasis, considered one of the hallmarks of cancers (1). This process involves the joint participation of endothelial cells, other stromal cells and the extracellular matrix (ECM) (2–4). Unlike normal tissue vasculature, the tumor vasculature exhibits abnormal structure and function, including a disordered vascular network, narrowed lumen, increased permeability and other defects, which leads to internal tumor hypoxia and low PH, inefficient drug delivery and reduced sensitivity to radiotherapy in the clinic (5–7).

Inhibition of angiogenesis is widely acknowledged as a promising antitumor strategy, exhibiting antitumor effects in various solid cancers. However, excessive inhibition of tumor angiogenesis tends to cause vascular degeneration, reduce drug delivery, and induce drug resistance (8, 9). In addition, treatment with single anti-angiogenic drugs tends to cause compensatory expression of other pro-angiogenic factors or pathway activation, leading to drug resistance (10–13). An increasing body of evidence from recently published studies suggests that short-term inhibition of tumor growth can induce normalization of tumor vascular structure and function, increase tumor vascular perfusion capacity while alleviating intratumor hypoxia levels, and improve the tumor suppressive immune microenvironment, which has the potential to potentiate the efficacy of radiotherapy and immunotherapy (14–16).

In recent years, nanomedicine has achieved significant progress, especially for targeted drug delivery and precision interventions (17, 18). Nanomedicines have unique advantages in terms of precision and multi-targeted interventions while showing enhanced effects when combined with other antitumor treatment strategies (19–21). This review provides a comprehensive overview of the characteristics and molecular basis of tumor vascular structure and function and describes the impact of tumor vascular abnormalities on the tumor immune microenvironment. Furthermore, we summarize the latest research advances of current anti-angiogenic nanomedicines and their effects on the immune microenvironment and immunotherapy.

## Regulation and characteristics of tumor vascularization

### The main processes and the molecular basis of tumor angiogenesis

Tumor angiogenesis is the biological process by which tumor cells and tumor microenvironment (TME) induce microvascular growth and establish blood circulation for tumor tissue (22). Tumor vasculature provides oxygen and nutrients for tumor tissues while carrying away metabolic wastes and carbon dioxide, promoting tumor growth and providing a pathway for tumor metastasis (23). It has been established that almost all solid tumors cannot grow beyond the size of 1-2 mm^3^ without the support of the vascular system, which indicates that neovascularization is a prerequisite for the sustained growth of tumors (24). Sprouting angiogenesis is the main and classical way of tumor angiogenesis and is predominantly discussed in this paper. This process refers to the generation of microvessels by endothelial cells in a “budding” manner based on the original blood vessels. The main steps include: ① degradation of vascular basement membrane and activation of endothelial cells; ② endothelial cell migration and proliferation; ③ endothelial cell formation of luminal structures and capillaries; ④ generation of new basement membrane and recruitment of pericytes to build vessel wall, formation of mature vessels and extension into solid tumors (25, 26).

Tumor angiogenesis is a complex process involving multiple mediators, mainly composed of tumor cells, tumor stromal cells (including endothelial cells, inflammatory cells, pericytes, fibroblasts, smooth muscle cells, etc.), extracellular matrix (ECM), and a variety of cytokines (27–29). All these effectors constitute the regulatory network of tumor angiogenesis. Current evidence suggests that the imbalance between pro- and anti-angiogenic factors is the key to initiating angiogenesis (30). Under physiological conditions, pro- and anti-angiogenic factors are in balance, and blood vessels remain quiescent and rarely form new branches. During tumor angiogenesis, the pro-angiogenic signals is hyperactivating, disrupting the balance and inducing angiogenesis (24, 31). Many factors contribute to the disruption of homeostasis, such as carcinogenic mutations, hypoxia, low pH, tumor-associated inflammation, recruitment of immune cells, and nutritional deficiencies. Targeted intervention of these factors is the key strategy for nano-targeted anti-vascular therapy.

Angiogenesis-stimulating factors represent a group of mediators, including growth factors, bioactive lipids, ECM degrading enzymes, cytokines, adhesion molecules and a variety of small molecule nucleic acids (**Table 1**). In recent years, the role of adhesion molecules in tumor angiogenesis has received increasing attention (32). Among the five major classes of adhesion molecules identified so far, it has been thought that the integrin family are the most important molecules for the process of angiogenesis by mediate cell-ECM adhesion (33).However, several other adhesion proteins, including vascular endothelial cell calcium adhesion protein (VE-cadherin), endothelial cell adhesion molecule 1 (PECAM1), and intercellular adhesion molecule 1 (ICAM1), vascular cell adhesion molecule-1 (VCAM-1), have been reported to mediating the adhesion of immune cells in tumor blood vessels, which is the key process for tumor immune cells to enter the stroma of tumor tissue and performing immunocidal functions (34–36). Understanding the mutual relationship among these regulatory molecules and their role in promoting angiogenesis is crucial for elucidating the mechanism of tumor angiogenesis and developing effective anti-angiogenic therapies.

**Table 1:** The sources and effects of the sources tumor vasculogenesis regulators.

Regulators	Cell types	Effects of regulators
VEGFA	Tumor cell, Macrophages, Neutrophils and MDSCs, Mast cells, Eosinophils, Treg cells, B cells, NK cells, Platelets, Pericytes, CAFs	Pro-angiogenic; induce EC proliferation, migration and survival, as well as ECM remodelling, to facilitate sprouting angiogenesis
FGF2	Tumor cell, Macrophages, Neutrophils and MDSCs, Eosinophils, B cells, Platelets, CAFs	
CXCL8	Macrophages, Mast cells, Eosinophils	
CXCL12	Macrophages, Platelets, CAFs	
PlGF	Macrophages	
VEGFC	Tumor cell, Macrophages	
IL-1β	Macrophages	
IL-6	Macrophages, Eosinophils	
IL-4	TH2 cells	Potentially pro-angiogenic by stimulating the alternative (M2-like) activation of TAMs
IL-17	TH17 cells	Pro-angiogenic by inducing CAFs to release CSF3, which recruits pro-angiogenic neutrophils
ANGPT1	Platelets, Pericytes	Potentially angiostatic
ECM components	Tumor cell, Pericytes	Promote EC survival and, possibly, proliferation; they may contriute to stabilization of TABVs
TNF	Macrophages, Mast cells	Potentially angiostatic under the influence of IFNγ and other TH1 cytokines
MMPs	Macrophages, Neutrophils and MDSCs, Mast cells, Eosinophils, B cells	Promote EC actication and migration
CXCL9	Macrophages	Potentially angiostatic under the influence of IFNγ and other TH1 cytokines
CXCL10	Macrophages	
CXCL11	Macrophages	
Endostatin	Platelets	Potentially angiostatic
THBS1	Platelets	Potentially angiostatic
PAI1	Platelets	Potentially angiostatic

### Structural and functional abnormalities of tumor vasculature

**Figure 1:**
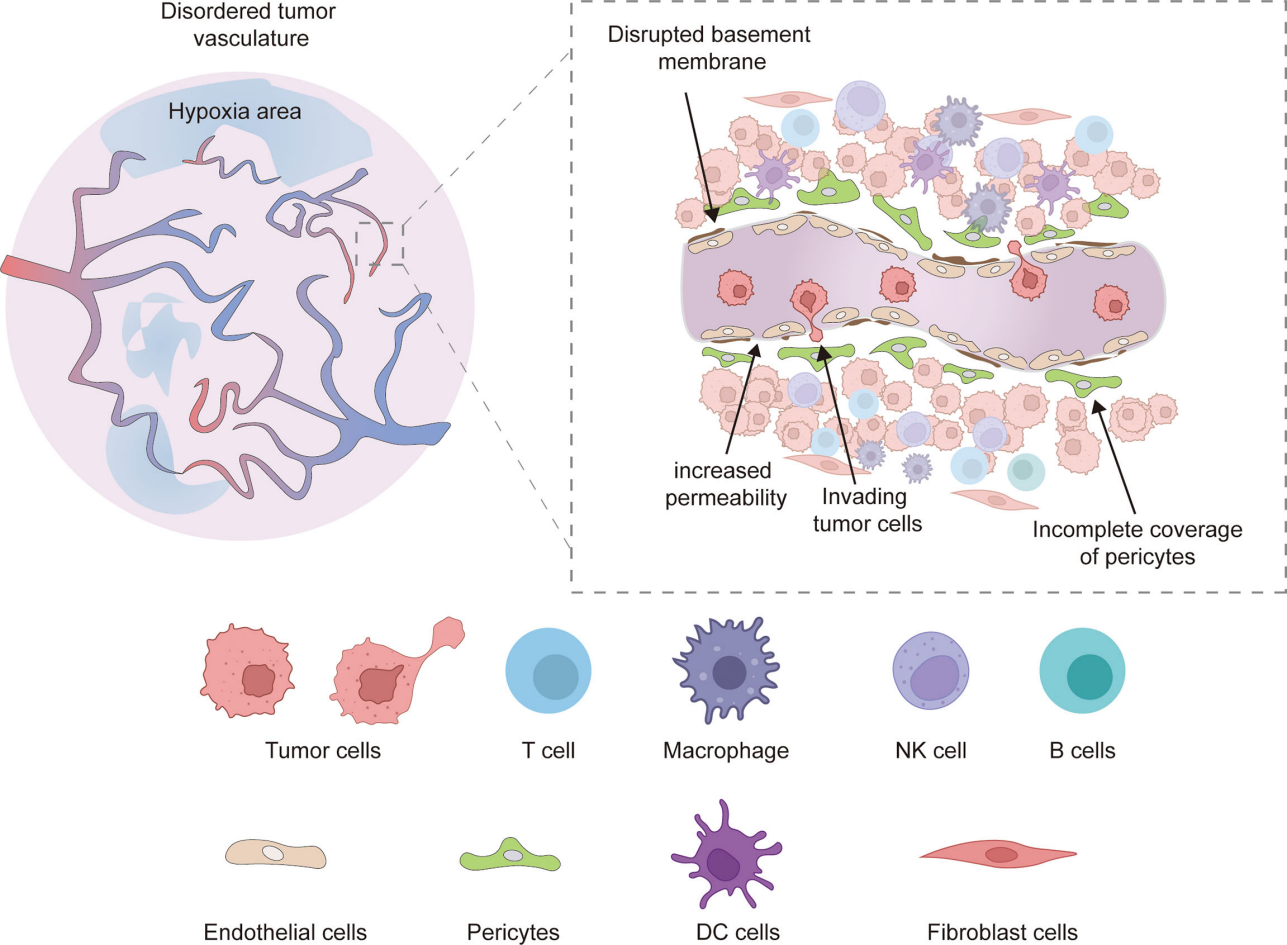
Abnormal tumor vessels are characterized as hyperplastic, tortuous, dilated, overlapping, and exhibit disordered branching, which leads to local tissue hypoxia. Besides, Defects in vascular branching, pericyte coverage, and basement membrane coverage were also observed in disordered tumor vasculature. The abnormal vessel structure and components increase vessel permeability, leading to increased tumor metastasis, and the tumor endothelial cells showed decreased adhesion molecules expression (ICAM1, VCAM1), resulting in reduced adhesion of immune cells on the surface of tumor vessels and infiltration in the tumor microenvironment. Hypoxia and low PH conditions also severely reduce the normal immune function of tumor immune cells.

Unlike physiological angiogenesis, tumor angiogenesis is a rapid and aberrant process, resulting in newly formed vessels that are often immature and have abnormal structures and functions (5). Tumor vessels are often hyperplastic, tortuous, dilated, overlapping, and exhibit disordered branching. The newly formed tumor microvessels lack normal structures, and are sinusoidal, striped, and thin-walled, composed of only one layer of endothelial cells. In addition, tumor vessels often exhibit high heterogeneity and distribution (**Figure 1**) (6, 14, 37).

It is widely thought that pericytes overlying endothelial cells play a regulatory role in the developmental stability and maturation of blood vessels. In tumor vessels, pericytes are detached from ECs, resulting in dysregulated flow characteristics and increased permeability. An incomplete vascular basement membrane (BM) is a feature of tumor vasculature. The BM of tumor vessels exhibits heterogeneity in thickness, fracture or absence and is loosely connected to endothelial cells and pericytes (15). In addition, TME leads to abnormal EC morphology and function. Compared with normal ECs, tumor-associated ECs change from a quiescent state to an activated state, showing high proliferation and migration, overlapping growth, loss of polarity, loose connections, and enlarged cell space (38). Meanwhile, the absence of a smooth muscle layer is also an abnormal indication of tumor vasculature (39). These structural and cellular abnormalities lead to abnormal vascular function; the neoplastic vessels exhibit lower blood flow, susceptibility to leakage, impaired vessel perfusion and increased vascular permeability, which lead to an abnormal TME: higher interstitial fluid pressure, hypoxia, acidosis and necrosis (40).In addition, restricted blood flow and high tumor interstitial pressure result in impaired drug delivery and reduced infiltration of immune effector cells (14). Meanwhile, hypoxia and acidic microenvironments impair the antitumor function of infiltrating immune cells (41). The abnormal vascular function makes tumors more susceptible to growth and metastasis and hinders the implementation of efficient tumor therapy.

## The effects of abnormal structure and function of the tumor vasculature on the tumor immune microenvironment

Immunotherapies such as immune checkpoint blockade are nowadays increasingly used for tumor treatment. The key to efficient immunotherapy is alleviating tumor immunosuppression and restoring the immune response (42). Tumor vascular abnormality is a major reason for the immunosuppressive TEM. As mentioned above, the abnormal structure and function of tumor blood vessels lead to interstitial hypertension and hypertonia and form a physical barrier, resulting in a reduced recruitment of immune effector cells (43). Besides, tumor-associated ECs express lower levels of adhesion molecules, such as intercellular adhesion molecule 1 (ICAM1), which prevents immune cells from adhering to endothelial cells, interferes with the trafficking of immune cells, and leads to a decrease in immune cell infiltration (especially T cell) (44, 45). Besides, upregulation of immune checkpoints such as PD-L1/2 in tumor-associated ECs leads to decreased recruitment and activation of cytotoxic T lymphocytes (CTLs) (46). It has been shown that Fas ligand (FasL), highly expressed in tumor-associated ECs, selectively mediates CTL but not Treg apoptosis (47).

Hypoxia is also a major driver that favors an immunosuppressive TME. Hypoxia causes immunosuppression *via* five mechanisms: (1) promoting the accumulation of immunosuppressive cells in tumor tissues, such as Treg, myeloid-derived suppressor cells (MDSCs), tumor-associated macrophages (TAMs), etc.; (2) promoting the production and release of immunosuppressive factors such as TGFβ, IL-10, Arg1, VEGFA (3) upregulating the expression of immunosuppressive molecules, such as PD-L1 on tumor cells, macrophages; PD-1 on CD8 + T cells; TIM and CTLA4 on MDSCs, Tregs, DCS and TAMs (4) inducing TAM to immunosuppressive M2 polarization phenotype which inhibits the maturation of DC cells and leads to the accumulation of immunosuppressive metabolites such as adenosine and lactate by changing the metabolism of tumor cells. (5) Attenuating survival, cytotoxicity and migratory activity of immune effector cells such as CD8 + cytotoxic T cells, CD4 + T cells and natural killer NK cells (48, 49). In addition, acidosis is a trigger for immunosuppression, as it reduces IFN-γ secretion by M1 macrophages and increases IL-1β secretion, inhibits T cell proliferation, promotes NK and T cell apoptosis, and inhibits CD8+ T cell and NK cell production of effector molecules (granzyme B, perforin, etc.). It also stimulates neovascularization by delaying the apoptosis of neutrophils and promoting infection (50, 51).

Excessive amounts of pro-angiogenic factors also contribute to the immunosuppressive TEM. For example, high-level VEGF promotes CTL depletion by upregulating the expression of immune checkpoint molecules (PD-1/PD-L1, CTLA4, LAG3, TIM3) (52). VEGF has also been associated with immune escape by inhibiting the differentiation and function of NK cells. In addition, VEGF triggers tumor evasion from the immune system by inducing Tregs and MDSCs proliferation (53). Growing evidence suggests that VEGF can promote M2 polarization of TAMs, inhibit the differentiation and maturation of DC cells as well as the antigen-presenting ability of mature DCs and reduce T cell infiltration (54, 55). As mentioned above, there is a complex interaction between the tumor vasculature and the tumor immune microenvironment. Normalizing the tumor vascular system increases immune cell infiltration and restores the antitumor capacity of immune cells, suggesting it is a potentially effective anti-cancer strategy (56).

## Targets of nanomaterial-based anti-angiogenic therapy

Angiogenesis represents an attractive target for cancer therapies because of the vital effects of tumor vessels in tumor progression. Due to the lack of specific biomarkers and resistance associated with a combination of the traditional approach with anti-angiogenic drugs, the efficacy during clinical practice remains unclear. Nanotechnology provides a new approach whereby nanomaterials bind to the potential target and release anti-angiogenic drugs at specific sites (57, 58). The nano drug delivery systems can reduce adverse effects on non-target healthy tissue, provide accumulation into the target tissue and protect nucleic acids, including small interfering RNA (siRNA) and micro RNA (miRNA), from rapid degradation in the body (59). Mechanisms and targets of nanomaterial-based anti-angiogenic therapy are as follows (**Table 2**).

**Table 2:** Mechanisms and targets of nanomaterial-based anti-angiogenic therapy.

Classifications	Nanoparticles	Tumour models	Targeted location	Mechanism	Effect	References	DOI
Liposome carriers	Single drug or triple drugs (paclitaxel, verteporfin, and CA4)-NP	Breast cancer	Tumor cells	Inhibit the Hippo/YAP (Yes-associated protein) pathway to inhibit CSCs,inhibit VEGFA/VEGFR and reduce HIF-α	Inhibit TNBC cell viability and cell migration, shorten/misshape intersegmental vessels, inhibit tumorsphere formation	A triple-drug nanotherapy to target breast cancer cells, cancer stem cells, and tumor vasculature	10.1038/s41419-020-03308-w
	Carvacrol-NP	Lung carcinoma	Tumor cells	Inhibit COX-2, MMP, CD31 and VEGF, decrease the activation of MAPK p38 as well as ERK and inhibit tumor cells migration	Inhibit tumor growth	Carvacrol encapsulated nanocarrier/ nanoemulsion abrogates angiogenesis by downregulating COX-2, VEGF and CD31 in vitro and in vivo in a lung adenocarcinoma model	10.1016/j.colsurfb.2019.06.016
	TPT/ICG@Lip-ERL NP	Breast cancer	Tumor cells	Inhibit EGFR, VEGF, and HIF-α	Inhibit endothelial tube formation, promote vessel maturation, decrease fibroblast expression, increase CD3+ T cell invasion, promote CD8+ and CD4+ T cells infiltration and inhibit tumor growth	Reshaping Tumor Blood Vessels to Enhance Drug Penetration with a Multistrategy Synergistic Nanosystem	10.1021/acs.molpharmaceut.0c00077
	Podo-NP and CbP-NP	Lung cancer	Tumor cells and endothelial cells	Downregulate VEGF and inhibit endothelial cell migration	Reduce microvessel density, decrease vessel leakage, induce tumor necrosis, decrease blood vessel length, enhancement antitumor immunity and inhibit tumor growth	Sequential Treatment of Bioresponsive Nanoparticles Elicits Antiangiogenesis and Apoptosis and Synergizes with a CD40 Agonist for Antitumor Immunity	10.1021/acsnano.0c07132
	SFN-LNC	Glioblastoma	Tumor cells and endothelial cells	Regulate RTKs and the RAF/MEK/ERK pathway, reduce Ki67+ cell and inhibit cell proliferation	Reduce tumor vessel area decrease tube formation increase vascular perfusion	Development and characterization of sorafenib-loaded lipid nanocapsules for the treatment of glioblastoma	10.1080/10717544.2018.1507061
	PTX/LGC-NP	Hepatocellular carcinoma	Tumor cells, endothelial cells and stromal cells	Inhibit VEGF, upregulate TSP-1 and induce cytotoxicity against tumor cells and endothelial cells	Reduce microvessel density, promote vessel maturation, reduce tumor vessel leakage and increase vascular perfusion	Attempts to strengthen and simplify the tumor vascular normalization strategy using tumor vessel normalization promoting nanomedicines	10.1039/c8bm01350k
Inorganic nanocarriers	Sorafenib and sgRNA-NP	Hepatocellular carcinoma	Tumor cells	Inhibit the EGFR-PI3K-Akt pathway	Inhibit tube-formation and tumor growth	Co-delivery of Sorafenib and CRISPR/Cas9 Based on Targeted Core-Shell Hollow Mesoporous Organosilica Nanoparticles for Synergistic HCC Therapy	10.1021/acsami.0c17660
	AuNPP-FA	Breast cancer and gastric tumor	Tumor cells	Promote the secretion of SEMA3A in tumor cells to inhibit Smad2/3 signaling in endothelial cells	Reduce tumor vessels, make tumor vessels become less chaotic and complex, promote vessel maturation, strengthen adherent junctions in endothelial cells, increase vascular perfusion and decrease permeability, increase in CD3+CD8+ T cell infiltration and inhibit tumor growth and metastasis	Gold Nanoparticles Induce Tumor Vessel Normalization and Impair Metastasis by Inhibiting Endothelial Smad2/3 Signaling	10.1021/acsnano.9b08460
	AuNP	Melanoma	Tumor cells and endothelial cells	Suppress the migration of tumor cells and endothelial cells, reduce MMP-2 and c-Myc and inhibit EMT	Reduce average vascular density, form more homogeneous and organized vascular morphology, increase vascular perfusion and decrease permeability	Gold nanoparticles attenuate metastasis by tumor vasculature normalization and epithelial–mesenchymal transition inhibition	10.2147/ijn.S128802
	New Sor-AuNP	Melanoma	Tumor cells and endothelial cells	Downregulate VEGF, MMP-2, c-Myc, SPARC, EGFR and VEGFR-2, inhibit tumor cell proliferation and reverse EMT	Reduce vessel density, enhance vessel perfusion, decrease vessel leakage and inhibit tumor growth and metastasis	Sorafenib derivatives-functionalized gold nanoparticles confer protection against tumor angiogenesis and proliferation via suppression of EGFR and VEGFR-2	10.1016/j.yexcr.2021.112633
	rhES-AuNP	Colorectal cancer	Endothelial cells	Decrease AGR2 expression, suppress AGR2-mediated cell migration, tube formation, and AGR2-induced activation of signaling pathway and reduce HIF-1α	Make vessels become organized and continuous, promote vessel maturation and improve vascular permeability	Conjugation of gold nanoparticles and recombinant human endostatin modulates vascular normalization via interruption of anterior gradient 2-mediated angiogenesis	10.1177/1010428317708547
	rhES-AuNP-PEG	Hepatocellular carcinoma	Endothelial cells	Downregulate HIF-1α	Promote vessel maturation, increase blood perfusion, decrease vessel leakage and inhibit tumor growth	Gold Nanoparticle-Mediated Targeted Delivery of Recombinant Human Endostatin Normalizes Tumour Vasculature and Improves Cancer Therapy	10.1038/srep30619
	CaBP-PEG	Breast cancer	Macrophages	Reduce macrophages from M2-type to M1-type and inhibit EGFR	Enhance vessel perfusion and inhibit tumor growth	Calcium Bisphosphonate Nanoparticles with Chelator-Free Radiolabeling to Deplete Tumor-Associated Macrophages for Enhanced Cancer Radioisotope Therapy	10.1021/acsnano.8b06699
Other carriers	GE11&GALA-pshVEGF@SNP	Lung cancer	Tumor cells	Deliver shRNA and inhibit VEGF	Reduce microvessel density	Multi-functional self-assembled nanoparticles for pVEGF-shRNA loading and anti-tumor targeted therapy	10.1016/j.ijpharm.2019.118898
	5-FU/siRNA@GalNAc-pDMA	Hepatocellular carcinoma	Tumor cells	Deliver siRNA, downregulate the expression of VEGF and VEGFR2 and inhibit tumor cells migration	Reduce the cell viability and induce cell necrosis	Inhibition of Metastatic Hepatocarcinoma by Combined Chemotherapy with Silencing VEGF/VEGFR2 Genes through a GalNAc-Modified Integrated Therapeutic System	10.3390/molecules27072082
	CQ-survivin-shRNA-NP	Lung cancer	Tumor cells	Inhibit EGFR, Dll4 and VEGF, block cell cycle and induce cell apoptosis	Reduce microvessel density, reduce tumor vessel permeability, promote vessel maturation and inhibit tumor growth	Chloroquine in combination with aptamer modified nanocomplexes for tumor vessel normalization and efficient erlotinib/Survivin-shRNA co-delivery to overcome drugresistance in EGFR-mutated NSCLC	10.1016/j.actbio.2018.06.034
	R300-doxorubicin-NP	Breast cancer and lung cancer	Tumor cells and platelets	Deplete intratumoural platelets, cause vascular damage, facilitate vascular permeability and enhance the efficacy of Dox.	Inhibit tumor growth and metastasis	Nanoparticle-mediated local depletion of tumour-associated platelets disrupts vascular barriers and augments drug accumulation in tumours	10.1038/s41551-017-0115-8
	Folate-heparin-NP	Ovarian cancer	Tumor cells and endothelial cells	Inhibit EMT and MMP2 and inhibit cell migration and invasion	Reduce microvessels and VM adn inhibit tumor growth	cRGD-functionalized nanoparticles for combination therapy of anti-endothelium dependent vessels and anti-vasculogenic mimicry to inhibit the proliferation of ovarian cancer	10.1016/j.actbio.2019.06.039
	siRNA-HA-CH-NP/siRNA-CH-NP	Ovarian cancer	Endothelial cells	Deliver siRNA and inhibit PLXDC1	Reduce microvessel density, inhibit cell proliferation and increase cell apoptosis	Selective delivery of PLXDC1 small interfering RNA to endothelial cells for anti-angiogenesis tumor therapy using CD44-targeted chitosan nanoparticles for epithelial ovarian cancer	10.1080/10717544.2018.1480672
	Sunitinib-NP	Kidney cancer	Endothelial cells	Target tyrosine kinases receptors	Reduce intratumoral microvascular density, inhibit tumor growth, inhibit cell proliferation and increase cell apoptosis	Carrier-Enhanced Anticancer Efficacy of Sunitinib-Loaded Green Tea-Based Micellar Nanocomplex beyond Tumor-Targeted Delivery	10.1021/acsnano.9b00467
	T4(the hydrophobic peptide(NLLMAAS))-NP	Breast cancer	Endothelial cells and macrophages	Inhibit Tie-2	Reduce vessel density, inhibit tumor growth and metastasis and induce cell necrosis	Cooperatively Responsive Peptide Nanotherapeutic that Regulates Angiopoietin Receptor Tie2 Activity in Tumor Microenvironment To Prevent Breast Tumor Relapse after Chemotherapy	10.1021/acsnano.8b08142
	G5-methotrexate-NP	Ovarian cancer	Macrophages	Reduce in VEGF-C, downregulate BRCA1 and BRCA2 gene expression to deplete TAMs and reduce CSCs	Reduce microvessel density and inhibit tumor growth	Therapeutic Impact of Nanoparticle Therapy Targeting Tumor-Associated Macrophages	10.1158/1535-7163.Mct-17-0688
	Cellax-DTX polymer	Pancreatic cancer	Fibroblasts	Reduce tumor-associated fibroblasts and macrophages	Inhibit tumor metastasis and increases tumor perfusion and survival	Targeting of metastasis-promoting tumor-associated fibroblasts and modulation of pancreatic tumor-associated stroma with a carboxymethylcellulose-docetaxel nanoparticle	10.1016/j.jconrel.2015.03.023
	TH10-DTX-NP	Lung cancer	Pericytes	Induce tumor vascular pericytes apoptosis	Reduce microvessel density, inhibit tumor cell proliferation and inhibit tumor metastasis	Selective eradication of tumor vascular pericytes by peptide-conjugated nanoparticles for antiangiogenic therapy of melanoma lung metastasis	10.1016/j.biomaterials.2013.12.027
	Doxorubicin-NP	Colon carcinoma	Protein	Inhibit VEGF165	Reduce microvessel density, enhance vessel perfusion and inhibit tumor growth	Sequestering and inhibiting a vascular endothelial growth factor in vivo by systemic administration of a synthetic polymer nanoparticle	10.1016/j.jconrel.2018.12.033

### Pro-angiogenic and anti-angiogenic factors secreted by tumor and immune cell

Vascular endothelial growth factor A (VEGFA) is a crucial regulator of angiogenesis progression. It binds to VEGFR2 in vascular endothelial cells to stimulate the proliferation and trigger endothelial cell migration through the RAS–RAF–MAPK (mitogen-activated protein kinase)–ERK (extracellular signal-regulated protein kinase) signaling pathway (27). Accordingly, VEGF gene expression regulation has attracted significant interest in anti-angiogenic cancer therapy. *In vitro* and *in vivo* experiments showed that plasmid DNA containing a small hairpin RNA (shRNA) expression cassette with epidermal growth factor receptor (EGFR)-specific binding ligand GE11 and pH-sensitive fusogenic peptide GALA could target tumor cells and induce endocytosis, whereby nanoparticles (GE11&GALA-pshVEGF@SNPs) entered tumor cells to inhibit VEGF expression, reduce tumor angiogenesis, and inhibit tumor growth (60). The RNA interference (RNAi) system based on gene silencing mechanisms is a powerful tool in antitumor angiogenic therapy. SiRNA has also been applied for the regulation of VEGF expression. Asialoglycoprotein receptor (ASGPR) is a mammalian lectin specifically expressed on the hepatocytes. N-acetyl-galactosamine (GalNAc) residues can mediate the specific cellular uptake of the nanoparticle by targeting ASGPR-expressing liver cells as one of the specific ligands for ASGPR. Due to the nanoparticles’ high compatibility and low cytotoxicity, they were modified with GalNAc to enter hepatocellular carcinoma, and siRNA was used to regulate VEGF transcription (61). However, the limitations of non-specific absorption and low efficiency of cellular uptake reportedly hamper their clinical applications. Fortunately, a method was proposed to tackle this problem by generating siRNA *in situ* through enzyme-free DNA amplification (62). Moreover, a new system containing superparamagnetic iron oxide nanoparticles, calcium phosphate (CaP) and PEG-polyanion block copolymers could improve the efficiency of siRNA transportation and promote its aggregation (63). In addition, blocking VEGF rather than influencing gene expression *in vivo* can be an anti-angiogenic approach. VEGFA165 is thought to be the most frequently expressed isoform in tumors and is the most physiologically relevant VEGFA isoform (27). Synthetic copolymer nanoparticles (NPs) were synthesized as the protein affinity reagents (PARs) that bind with vascular endothelial growth factor (VEGF165) to inhibit angiogenesis *in vivo (* 64)

However, anti-angiogenic therapy through inhibition of the VEGF pathway has its limitations. In this respect, no Phase III clinical trial has shown anti-angiogenic benefits during glioblastoma therapy. Clavreul et al. considered it might be attributed to VEGF-independent angiogenesis, induction of tumor invasion and inefficient antiangiogenic factor delivery to the tumor (65). CD11b+Gr1+ cells, including neutrophils, macrophages, and myeloid-derived suppressor cells, promote growth factor granulocyte colony-stimulating factor (G-CSF) and promote myeloid cell-dependent angiogenesis *via* secreting protein Bv8. This angiogenic pathway bypassing VEGF represents the response mechanism to anti-VEGF therapy (65). Besides, cancer stem cells (CSCs) can drive tumor drug resistance. Interestingly, CSCs are a small subpopulation of cells with stem cell-like characteristics that can self-renew, divide asymmetrically to give rise to daughter cells and modulate the vascularization of the tumor by promoting the expression of HIF-1, VEGF and SDF-1/CXCL12 (66). Antivascular therapies are known to drive CSCs propagation and lead to the appearance of resistance mechanisms (67). Therefore, reducing CSCs in antivascular therapy can be applied in nanomaterial-based anti-angiogenic therapy. In triple-negative breast cancer (TNBC), encapsulation of paclitaxel, verteporfin, and combretastatin (CA4) significantly reduces CSCs and gene expression of VEGFA as well as the VEGF receptor (68). In ovarian cancer, tumor-associated macrophages can be selected by binding G5-MTX nanoparticles with folate receptor-2(FOLR2) which is highly expressed in TAMs. These nanoparticles can overcome resistance to anti-VEGF-A therapy inhibition of VEGF-C and BRCA1 gene expression for depletion of TAMs and the reduction of CSCs. Also, through coculture of TAMs and tumor cells, a mild increase in sphere number of tumor cells suggested that TAMs promoted stemness. In other words, depleting TAMs could reduce stemness (69).

Besides the VEGF pathway, multiple signaling pathways are related to nanomaterial-based anti-angiogenic therapy. It has been reported that the EGFR-PI3K-Akt pathway can be regulated at the gene level through CRISPR/Cas9 systems with a chitosan nanodelivery system to recognize the HepG2 cell adhesion molecules (CAMs) during the treatment of hepatocellular carcinoma. Tube formation of HUVEC cells was significantly inhibited after NPs treatment *in vivo (* 70). In addition, it has been reported that Tyrosine kinase with immunoglobulin and epidermal growth factor homology-2(Tie-2) is expressed in endothelial cells, and tie-2 positive macrophages (TPM) promote tumor vascular reconstruction and chemoresistance through the angiopoietin (ANG)/Tie2 signaling pathway. Researchers designed dual-responsive amphiphilic peptide (mPEG1000-K(DEAP)-AAN-NLLMAAS) to modify the small peptide T4 (NLLMAAS) to protect T4 and target the acidic tumor microenvironment. T4 released by the ultimate nanoformulation (P-T4) could interact with tie-2 to inhibit the phosphorylation of downstream molecule focal adhesion kinase, which is relevant to migration and tubule formation (71).

### Pro-angiogenic and anti-angiogenic signaling pathways in vascular endothelial cells

As mentioned above, tumor endothelial cells (TEC) have characteristics that lead to leakiness and chaotic blood flow of tumor vessels. There are different markers, including EGFR, Pax2 and CXCR7 in TEC, which can bind with numerous ligands, such as peptides, nucleic acids aptamers, sugars and cationic charged materials. These markers can be harnessed to distinguish from normal endothelial cells and target TEC (72, 73). Nanomaterials can be loaded with antiangiogenic drugs with different effects. Accordingly, target tumor vessel therapies can be classified into two categories: anti-angiogenic agents (AAs) and vascular disrupting agents (VDAs) (74).

As for the target of vascular endothelial cells, the VEGF signaling pathway has drawn much attention. Tumor endothelial marker 7(TEM7), referred to as PLXDC1, has a plexin-semaphorin-integrin (PSI) domain. The PSI domain exists in semaphorin, and its receptor acts as a vascular endothelial growth factor (VEGF) receptor in endothelial cells (75). In a recent study, tumor endothelial cell CD44 receptor was specifically targeted by hyaluronic acid (HA), and angiogenic gene PLXDC1 was silenced by siRNA by combining with chitosan (CH) for endocytosis. Thus HA-chitosan- nanoparticle/siRNA system provides a new approach for anti-angiogenesis tumor therapy at the transcriptional level (76).

Besides, interleukin-8 (IL-8) is another pro-angiogenic factor activated by the VEGF pathway. It plays a role in the tube formation of endothelial cells. Poly (ethylene glycol)-conjugated epigallocatechin-3-O-gallate (PEG-EGCG) loaded with sunitinib has been reported to gather in tumor tissue. Moreover, Sunitinib-loaded micellar nanocomplex (SU-MNC) inhibits the mRNA and protein expression of IL-8 in endothelial cells to inhibit proliferation and migration (77).

Multimodal antivascular nanodevice of AAs and VDAs for cancer therapy has been reported to maximize the therapeutic potential of antivascular therapeutics. Some nanodevices have additional anti-angiogenic capabilities, such as photothermal therapy (PTT), which create local high-temperature conditions *via* gold nanorod and photodynamic therapy (PDT), producing toxic reactive oxygen species *via* photosensitizers under near-Infrared light conditions (78, 79). Moreover, the precise localization characteristics of nanoparticles promote the efficiency of PTT and PDT. The ferritin nanocage (Fn) targeting CGKRK peptides in the tumor loaded with “556-Ph”, a new type of metalla-aromatics complex of NIR-absorbing organic agent, yielded a better performance with PTT and PDT (80). Furthermore, combining PTT, PDT and VDA to destroy blood vessels can achieve better results. Nanotechnology can be used to develop a vascular disrupting agent that can be released before anti-angiogenic agents. VDAs can decrease the number of vessels by destroying the tumor’s neo-formed vasculature and ensure nanoparticles can more efficiently target specific regions (67).

### Other targets of nanomaterial-based anti-angiogenic therapy

Matrix metalloproteinases (MMPs), abundant in the tumor extracellular matrix (ECM), can decompose the extracellular matrix and disrupt the tumor microenvironment to promote the metastasis of cancer cells and induce angiogenesis. Moreover, they can biologically activate VEGF and induce endothelial cell motility (81). Therefore, MMPs are also regarded as another potential therapeutic target. Paclitaxel (PTX) nanocrystals were prepared with MMP-sensitive β-casein/marimastat (MATT) and PTX to treat breast cancer metastasis. These complexes could target tumor cells and the local tumor microenvironment and suppress the activities of MMP-2 and MMP-9 *in vivo (* 82). In lung cancer, integrating chemotherapy drug carriers with Versatile polypeptide-LinTT1-Pvgli-Tat (LPT) increased tumor-penetrating capacity, reduced MMPs, inhibited tumor growth and decreased blood vessel density (83). A new nanomaterial called carvacrol nanoemulsion (CN) yielded anti-angiogenesis effects at multiple stages. CN influenced COX-2 to decrease MMPs and VEGF levels and could bind with the glycine residue of VEGF and the allosteric area of CD31 to disrupt adhesion between endothelial cells. In addition, CN could block MAPK and NF-κb pathways to interfere with the effect of VEGF and suppress inflammation to reduce angiogenesis, respectively (84).

Platelet aggregation is induced by tumor cells, tumor-infiltrating macrophages, and tumor endothelial cells by activating the coagulation pathway. Tumor-derived ADP, cathepsin B, and matrix metalloproteinases also play a crucial role. Furthermore, planets promote angiogenesis through microRNA, lipids, platelet-derived microparticles, and surface receptors (85). Recent reports suggest that platelets act as a membrane in nanoparticles and prevent tumor-specific immunological recognition (86). On the other hand, nanoparticles loaded with platelet inhibitors have been designed for tumor therapy. For example, a new nanoparticle coated with MMP2-cleavable peptides for targeted aggregation of MMP2 in tumor tissue contains R300 that can bind to platelet receptors and deplete platelets (87). Moreover, it can be used to increase drug permeability. However, more emphasis should be placed on bleeding complications in blocking specific tumor-platelet interaction sites (88).

Pericytes, perivascular cells associated with resistance to anti-VEGF therapeutics, play a critical role in tumor angiogenesis. Guan et al. developed new nanoparticles (TH10-DTX-NP) conjugated with TH10 peptide (TAASGVRSMH) and loaded with docetaxel. TH10 peptide could bind with NG2 receptors, highly expressed on the pericytes, and DTX could induce pericyte apoptosis. Consequently, it was shown that TH10-DTX-NP-induced pericyte apoptosis could decrease microvessel density and inhibit metastases in melanoma therapy (89).

Over recent years, it has been found that vasculogenic mimicry (VM) of tumor cells, an alternative circulatory system, plays a crucial role in tumor angiogenesis. Highly aggressive tumor cells can form vessel-like structures by mimicking endothelial cells. These structures can also provide sufficient blood supply and nutrients to tumors (90), providing an effective nanomaterial-based anti-angiogenic strategy. Moreover, CRGD was conjugated with heparin and folic acid to form two nanoparticles (CRGD-NPS1 and CRGD-NPS2). CRGD-NPS2 could regulate the MMP2/Laminin 5c2 signal pathway to inhibit endothelium-dependent vessels (EDV) and tumor cell-mediated VM in selected ovarian cancer cells capable of forming VM channels (91).

## Nanomaterials with tumor vascular-targeting properties promote the normalization of tumor vasculature

### Tumor vasculature-targeting nanomaterials induced normalization of tumor vasculature

**Figure 2:**
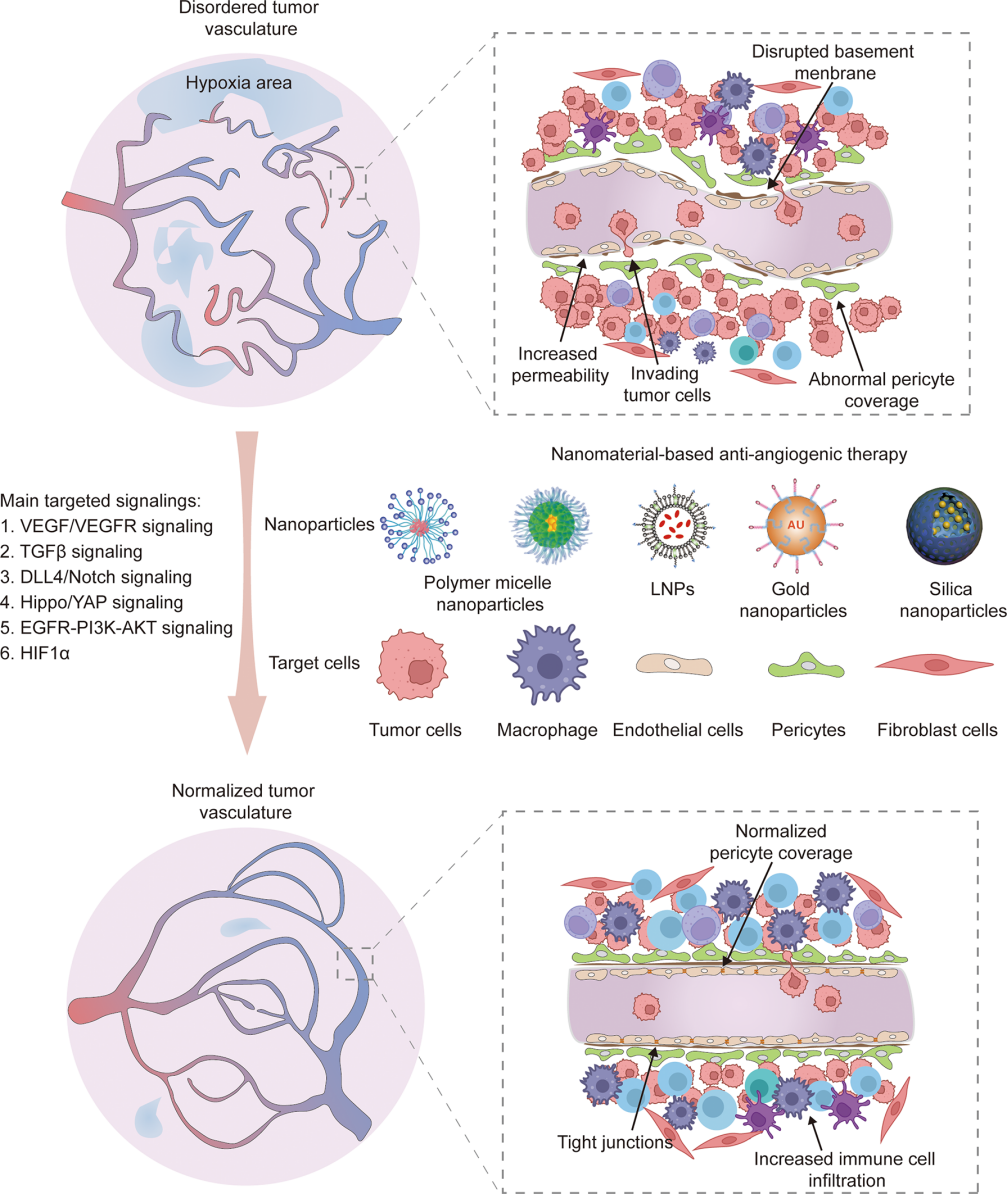
The normalization of tumor vasculature restores normal vessel function like decreasing micro-vessel density, improving pericyte coverage, increasing endothelium tight junctions and promoting immune cell infiltration. Various nanoparticle carriers including polymer micelle nanoparticles, lipid nanoparticles (LNPs), gold nanoparticles and silica nanoparticles have been applied to target various cell types, such as tumor cells, macrophages, endothelial cells, pericytes and fibroblast cells. The main targeted signaling are VEGF/VEGFR, TGFβ-Smads, DLL4/Notch, Hippo/YAP, EGFR-PI3K-AKT and HIF1α.

Normalization of tumor vasculature reportedly improves the effect of antitumor therapy by correcting structural abnormalities of tumor vessels and restoring normal function to a certain extent. This finding suggests that sustaining the balance between pro-angiogenic and anti-angiogenic factors is essential to decreasing micro-vessel density, improving pericyte coverage and regulating vascular permeability and perfusion. As a result, normal vessels relieve hypoxia to inhibit tumor invasion and recurrence (**Figure 2**). Moreover, vascular normalization is conducive to subsequent treatment, including chemotherapy (92), radiotherapy (93) and immunotherapy (94). Importantly, nanotechnology offers an excellent platform to improve the bioavailability and stability of drugs, reduce side effects and achieve combination therapy. Hence, various nanomedicines have been applied to target abnormal tumor vessels for more efficient therapy (95).

Epithelial-mesenchymal transition (EMT) is a process by which epithelial cells lose their adhesiveness and gain increased motility, contributing to VM formation and tumor cell metastasis. It can be stimulated by many factors, including c-myc, MMP-2 and HIF-α (96). Over the years, gold nanoparticles(AuNPs) have been applied in nanomaterial-based vascular normalization therapy of breast cancer (97), melanoma (96, 98), liver cancer (99) and metastatic colorectal cancer (100). Intriguingly, AuNPs could inhibit MMP-2 and c-Myc to suppress EMT and the migration of HUVECs (96). Fan Pan et al. conjugated gold nanoparticles and recombinant human endostatin to modulate vascular normalization in metastatic colorectal cancer. They found that NPs inhibited EMT *via* interruption of anterior gradient 2 (AGR2)-mediated angiogenesis, a latent tumor angiogenesis factor regulated by hypoxia-induced factor-1 (HIF-1) (100). Changes in tumor vessels after AuNPs vascular normalization therapy include a decrease in vascular density, permeability and hypoxic area vascular density and increased vascular perfusion, pericyte coverage of tumor and α -smooth muscle actin (α -SMA), an index of vessel maturation (96–100).

It is well-established that TAMs play a pivotal role in tumor angiogenesis through VEGF and other pathways, which leads to abnormal tumor vasculatures (101). TAMs are generally classified into immunosuppressive M2-type and immunosupportive M1-type. M2-type macrophages represent the dominant type of TAMs related to tumor growth, progress and metastasis. Furthermore, myeloid cell infiltration and differentiation are triggered by a hypoxic microenvironment; thus, more TAMs are recruited to the tumor site, which leads to poor therapeutic outcomes (102). Tian et al. designed calcium bisphosphonates (CaBP-PEG) nanoparticles that gather in tumor tissue through enhanced permeability and retention (EPR). In breast cancer treatment, NPs reduced macrophages from M2-type to M1-type and blocked the TAMs-stimulated angiogenesis signaling pathway. The dilation of tumor blood vessels and saturated oxygen indicated normalization of tumor vasculature and relief from hypoxia (103).

### Other targeted nanomaterials induce normalization of tumor vasculature

Current evidence suggests that tortuous and highly permeable tumor vessels cause accumulation of vascular macromolecules in tumor tissues and high interstitial fluid pressure (IFP), which prevents drug penetration (104). Furthermore, high IFP can aggravate the abnormal vascular network and hypoxia environment (105). Sirin Yonucu et al. demonstrated that tumor vascular normalization could restore intravascular pressure gradients and prune non-functional vessels by building a mathematical model and analyzing it with cases. Peripheral aggregation of drugs as micelles, nanoprobes and liposomes was changed into aggregation in the tumor center (104). Hence, vascular normalization and regulation of IFP decrease tumor repopulation caused by heterogeneous drug accumulation and increase the probability of treatment success. Hyaluronic acid (HA), a key component in ECM, is highly expressed in tumor tissue. Hyaluronidase (HAase) has been used to decompose HA and decrease IFP to enhance chemotherapy drug penetration for many years (106). Jingjing Jiao et al. designed the nanoparticle (PM@HAase-mPEG), combining a porphyrin-based metallacage, HAase and DSPE-mPEG2000 to normalize blood vessels, relieve hypoxia and promote the cellular accumulation of drugs. The results indicated that PM@HAase-mPEG treatment could increase the density of blood vessels, blood perfusion and oxygen content. In addition, normalizing tumor vasculature *via* HAase could enhance the efficacy of PDT therapy for breast cancer (105).

Tumor-associated fibroblasts (TAFs), derived from the “activation” of tumor stromal cells such as fibroblasts, can create tension in matrix components such as collagen. Mechanical vascular compression contributes to the abnormal structure of tumor vessels and hindrance of drug penetration (107). Moreover, depletion of TAFs has been shown to decompress vessels and improve perfusion (108).

Interestingly, nanotechnology can be harnessed to promote long-term blood circulation and high accumulation of drugs in tumor tissue. Mark J. Ernsting et al. designed Cellax-DTX polymer, a conjugate of docetaxel (DTX), polyethylene glycol (PEG), and acetylated carboxymethylcellulose. Cellax-DTX reduced CAF content and increased duct luminal area and blood perfusion during the treatment of pancreatic ductal adenocarcinomas (109). Furthermore, TAF-targeted normalization of tumor vasculature could improve nanomaterial-based tumor drug delivery. Celecoxib, a selective COX-2 inhibitor, was designed with PTX-loaded micelles to reduce TAFs and normalize tumor vessels. The mechanism of TAF depletion involved suppression of fibroblast proliferation and activation stimulated by FGF-2 and transforming growth factor beta-1 (TGF-beta1) by inhibiting ERK1/2 phosphorylation, inhibition of the CXCL12/CXCR4 axis, induction of G1-S cell cycle arrest and apoptosis of TAF and reprogramming of TAF into normal fibroblasts. Consequently, microvessel density and increased pericyte coverage on endothelial cells was reduced after lung cancer treatment. Furthermore, it increased the *in vivo* delivery of micelles and improved the therapeutic benefits of PTX-loaded micelles (110). The interaction of nanomaterials and vascular normalization demonstrates that their combination may have great potential to improve tumor treatment.

## The synergistic effects of nanomedicine induced vascular normalization in tumor immunotherapy

The abnormal structure and function of tumor vessels disrupt the tumor immune microenvironment. On the one hand, abnormal tumor vessels decrease antitumor infiltrating lymphocytes *via* high IFP, downregulation of adhesion molecules and hypoxia, which activates inhibitory signaling pathways for antitumor immune response. On the other hand, the hypoxic tumor microenvironment can recruit Treg and promote TAM polarization to immunosuppressive M2-like phenotype to participate in immune evasion (94). Moreover, normalization of tumor vasculature is beneficial in rescuing these immunosuppressive outcomes. Changes in immune cell infiltration in nanomaterial-induced tumor vascular normalization include increased levels of tumor-infiltrating CD4+ and CD8+ T lymphocytes, natural killer cells and dendritic cells, decrease in myeloid-derived suppressor cells and polarization of TAMs from M2-to-M1 type (97, 111–114). Immune regulatory changes may be attributed to the direct effects on immune cells, including depletion of M2-type macrophages, indirect effects when they induce changes in protein expression on endothelial cells or tumor cells and rescue hypoxia and physical effects through the morphology of vascular normalization or the reduction of ECM (115). Lenvatinib, an inhibitor of VEGFRs, was loaded in Bi/Se nanoparticles to promote tumor vascular remodeling. The anti-angiogenic drug Lenvatinib was reported to enhance memory T-cell recruitment and CD8+ T lymphocyte, which can be potentiated by Bi/Se nanoparticles (111). Furthermore, the expression of TGF-β genes involved in the ECM components synthesis was inhibited by tranilast combined with Doxil nanomedicine to induce tumor vessel normalization. The distances between cancer-associated fibroblasts and CD3+ T cells were measured to reflect collagen barrier size. This finding represents another way to shape the tumor microenvironment and restrict T cell infiltration (114).

The interactions between anti-angiogenic therapies and immunotherapies could be considered a ‘two-way street’. A series of preclinical and clinical studies indicated the mutually improved effect of anti-angiogenic and immune-checkpoint inhibitors (94, 115). Of course, only if the action of anti-angiogenic drugs are limited directly to the tumor with little influence on normal tissues will disrupting tumor vasculature be beneficial for immunotherapy. Therefore, nanotechnology is needed to prevent negative effects on healthy non-target tissue while providing accumulation into the target tissue (116). In addition, the nanomedicine-mediated PDT and PTT can promote dendritic cell maturation and immune cell activation. It is reported that PDT and PTT synergetic with immune therapy for cancer treatment (117, 118). Furthermore, nanoparticles can be created to enhance cooperative and coordinated therapeutic effects by co-delivery and targeting of many therapeutic agents, as well as through immunomodulation. Nanoparticles have the potential to enhance treatment results by assuming these functions in combination therapy techniques (119). It is known that the immune checkpoint molecule programmed cell death protein 1(PD-1) can bind with its ligand (PD-L1) to protect tumor cells from immune surveillance *via* T cell exhaustion. CD8+ T cell infiltration plays a key role in anti-PD-1/PD-L1 therapy (112). Combining tumor vascular normalization with anti-PD-L1 therapy in breast cancer (97, 114) and hepatocellular carcinoma (112) has attracted significant interest in recent years. As a result, combination therapies could increase CD3+CD8+T cells, inhibit tumor proliferation and prevent tumor metastasis, providing a promising strategy to enhance immune-checkpoint inhibitor efficacy and the antitumor immunity of highly metastatic tumors.

Vascular normalization can even regulate “cold” immune microenvironment and provide a platform for sequential immunotherapy. The CD40 agonist stimulates antigen-presenting cells to process tumor-associated antigens produced by dying tumor cells, reversing immunosuppressive tumor microenvironments. However, CD40 may not have significant anticancer efficacy as a single treatment in patients with immunologically “cold” tumor (120). Xiang Ling et al. created the combination of anti-angiogenesis bioresponsive nanoparticles and chemotherapy regimens with CD40 agonist in a lung cancer mouse model. Compared with single treatment, combination therapies can reverse immunosuppression to elicit strong systemic antitumor immunity (113). For immunosuppressed tumors, nanomedicine induced vascular normalization provides a new idea for the application of immunotherapy.

## Conclusion and prospects

Since the antitumor angiogenesis therapy was first proposed, the structure and function of the tumor microvasculature have gradually been revealed in the past two decades, along with advances in research tools and imaging techniques, and significant progress has been made in understanding the key mechanisms of angiogenesis and vascular remodeling in solid tumors, especially those involved in tumor angiogenesis and tumor vascular network maturation, and the effects of these structures on other components of the TME. The close interactions between these structures and other components of the TME are essential for the development of novel anti-angiogenic drugs and better guidance of combination antitumor therapy.

In recent years, immunotherapy has been widely studied and yielded promising therapeutic effects in preclinical studies. However, clinically, some patients do not respond to immune checkpoint therapy or are resistant to long-term use. It should be borne in mind that long-term use of immunotherapy treatment also presents serious cardiovascular side effects, etc. Achieving more efficient targeted delivery and improving suppressive TME while reducing toxic side effects warrants further research. Indeed, the past decade has witnessed unprecedented inroads in nanomedicine with the development of effective delivery systems that are not limited to targeting tumor cells but also normalize tumor vascular structure and function, reduce intratumor hypoxia, and increase blood perfusion while increasing immune cell infiltration and activating immune cells to achieve better immune functions in the TME.

Overall, the abnormalities of tumor vascular structure and function and its effects on the tumor microenvironment were summarized in this review, and the application of nano-strategies in combination with multi-targets and multi-combination nano-drugs showed significant anti-cancer effects in a wide range of solid tumors with low systemic toxicities, while exhibiting more low-dose and efficient antitumor effects when combined with other chemotherapeutic agents and immunotherapy.

However, several important issues remain to be addressed before anti-angiogenic nanodrugs are translated into clinical applications. First, the efficacy and safety of anti-angiogenic nanodrugs have only been evaluated over a relatively short period (a few days or weeks) in most current studies. Indeed, long follow-up observations and toxicological experiments are needed to ensure the absence of local or systemic toxic side effects of specific nanodrugs and other components before clinical translation. Currently available functional identification methods for tumor vascular normalization are only applicable in animals, and the establishment of reliable biomarkers and assays for vascular normalization for clinical practice is of great significance to refining the applicability of nanomedicines to cancer patients. Most current nanomedicines have been evaluated using allogeneic transplantation tumor models, and the application of spontaneous tumor models or PDX models would be more beneficial for evaluating the translational application value. Besides, quality control and the dose and mode of administration of nanomedicines warrant further evaluation in clinical trials. In addition, based on the series of issues mentioned above, almost all nanomedicines have failed to achieve the expected efficacy in antitumor clinical trials and clinical applications. More than 100 antitumor clinical trials of nanomedicines have been registered in clinical trials(https://clinicaltrials.gov/), including those based on LNP, Polysiloxane Gd-Chelates based nanoparticles (NCT04881032), Ceramide NanoLiposome (KNAN2001), SNB-101 (NCT04640480) and others. However, until now, only paclitaxel albumin-stabilized nanoparticle formulation has shown increased percentage of patients with response in clinical trials, but it also showed increased serious and other adverse events, such as hemoglobin decreased, neutrophil count decreased (NCT00626405). Therefore, there are still many issues that need to be thought and solved to achieve clinical translation of nanomedicines.

Despite these limitations, it is widely expected that the rapid development of nanomedicine will solve and greatly improve these problems in the next 5-10 years, and nanomedicines are expected to become the first or second line of standardized treatment for cancer patients.

## Author contributions

All authors contributed to writing and revising the manuscript content.

## Funding

This work is supported by grants from the National Natural Science Foundation of China (81871874 to KW), the Zhejiang Provincial Department of Science and Technology of China (2020C03027 to KW), the Key Science and Technology Projects of Jinhua Science and Technology Bureau(2022-3-047 to ZX).

## Acknowledgments

We are grateful to all the participants who have made this research possible. And we would like to thank Freescience for English language editing.

## Conflict of interest

The authors declare that the research was conducted in the absence of any commercial or financial relationships that could be construed as a potential conflict of interest.

## Publisher’s note

All claims expressed in this article are solely those of the authors and do not necessarily represent those of their affiliated organizations, or those of the publisher, the editors and the reviewers. Any product that may be evaluated in this article, or claim that may be made by its manufacturer, is not guaranteed or endorsed by the publisher.

## Glossary

**Table:**

AAs	anti-angiogenic agents
AGR2	anterior gradient 2
ASGPR	asialoglycoprotein receptor
AuNPs	gold nanoparticles
bFGF	basic fibroblast growth factor
BM	basement membrane
CAMs	cell adhesion molecules
CH	chitosan
CN	carvacrol nanoemulsion
COX-2	yclooxygenase-2
CSCs	cancer stem cells
CTLs	cytotoxic T lymphocytes
DTX	docetaxel
ECM	extracellular matrix
EDV	endothelium-dependent vessels
EMT	epithelial&ndash;mesenchymal transition
EPR	enhanced permeability and retention
ERK	extracellular signal-regulated protein kinase
FasL	fas ligand
GalNAc	N-acetyl-galactosamine
G-CSF	granulocyte colony-stimulating factor
HA	hyaluronic acid
HAase	hyaluronidase
HIF-1	hypoxia-inducible factor-1
ICAM1	intercellular adhesion molecule 1
ICIs	immune checkpoint inhibitors
IFP	interstitial fluid pressure
IL-8	interleukin-8
MAPK	mitogen-activated protein kinase
MDSCs	myeloid-derived suppressor cells
miRNA	micro RNA
MMP	matrix metalloproteinase
NPs	nanoparticles
PARs	protein affinity reagents
PD-1	programmed cell death protein 1
PDGF	platelet-derived growth factor
PD-L1	programmed cell death 1 ligand 1
PDT	photodynamic therapy
PECAM1	endothelial cell adhesion molecule 1
PEG	polyethylene glycol
PIGF	placenta growth factor
PSI	plexin-semaphorin-integrin
PTT	photothermal therapy
PTX	paclitaxel
RNAi	RNA interference
shRNA	small hairpin RNA
siRNA	small interfering RNA
TAFs	tumor-associated fibroblasts
TAMs	tumor associated macrophages
TEC	tumor endothelial cells
TGF-β	transforming growth factor beta
TKI	tyrosine kinase inhibitor
TME	tumor microenvironment
TNBC	Triple-negative breast cancer
TPM	tie-2 positive macrophage
VDAs	vascular disrupting agents
VE-cadherin	vascular endothelial cell calcium adhesion protein
VEGF	vascular endothelial growth factor
VEGFA	vascular endothelial growth factor A
VEGFR	vascular endothelial growth factor receptor
VM	vasculogenic mimic
α-SMA	α-smooth muscle actin
